# The Truth of Sensational Paragraphs

**Published:** 1897-08-28

**Authors:** 


### The Truth of Sensational Paragraphs.

Our readers may have noticed a paragraph in the daily papers with a sensational heading referring to injury to hospital patients. This appeared very formidable, but a visit to St. Bartholomew's Hospital showed that the accident amounted in reality to the falling of a small piece of plaster from the ceiling of the portico leading to the out-patients' room, which slightly injured two persons. One was bruised, and returned home, and one, a girl, had a little cut on her forehead. This cut was dressed in the surgery, and the patient returned home the next morning. News is scarce, and the silly season is responsible for the conjuring up of many sensational incidents such as the one we refer to, which vanish into thin air under the light of inquiry.

